# Epigenetic consequences of hormonal interactions between opposite‐sex twin fetuses

**DOI:** 10.1002/ctm2.234

**Published:** 2020-12-04

**Authors:** Siming Kong, Yong Peng, Wei Chen, Xinyi Ma, Yuan Wei, Yangyu Zhao, Rong Li, Jie Qiao, Liying Yan

**Affiliations:** ^1^ Center for Reproductive Medicine Department of Obstetrics and Gynecology Peking University Third Hospital Beijing China; ^2^ National Clinical Research Center for Obstetrics and Gynecology Beijing China; ^3^ Key Laboratory of Assisted Reproduction (Peking University) Ministry of Education Beijing China; ^4^ Beijing Key Laboratory of Reproductive Endocrinology and Assisted Reproductive Technology Beijing China; ^5^ Beijing Advanced Innovation Center for Genomics Beijing China; ^6^ Peking‐Tsinghua Center for Life Sciences Peking University Beijing China; ^7^ Research Units of Comprehensive Diagnosis and Treatment of Oocyte Maturation Arrest Beijing China; ^8^ Academy for Advanced Interdisciplinary Studies Peking University Beijing China

**Keywords:** DNA methylation, histone modification, opposite‐sex twins, twin testosterone transfer hypothesis

## Abstract

Previous studies reported inconsistent evidence about some phenotypic traits of females in human opposite‐sex twins (opposite‐sex females [OSF]) being distinct from females in same‐sex twins (SSF). Comparatively, less evidence showed significant differences between males in OS twins (opposite‐sex males [OSM]) and males in same‐sex twins (SSM). The twin testosterone transfer hypothesis suggests that prenatal exposure of testosterone *in* *utero* may be a possible explanation for the differential traits in OSF; however, the underlying mechanism is unknown. Here, we investigated the potential epigenetic effects of hormone interactions and their correlation to the observed phenotypic traits. In the study, DNA methylomic data from 54 newborn twins and histone modification data (H3K4me3, H3K4me1, H3K27me3, and H3K27ac) from 14 newborn twins, including same‐sex females (SSF), OS twins, and same‐sex males (SSM) were generated. We found that OSF were clearly distinguishable from SSF by DNA methylome, while OSM were distinguishable from SSM by H3K4me1 and H3K4me3. To be more specific, compared to SSF, OSF showed a stronger correlation to males (OSM and SSM) in genome‐wide DNA methylation. Further, the DNA methylomic differences between OSF and SSF were linked to the process involving cognitive functions and nervous system regulation. The differential H3K4me3 between OSM and SSM was linked to immune responses. These findings provide epigenetic evidence for the twin testosterone transfer hypothesis and offer novel insights on how prenatal hormone exposure *in utero* may be linked to the reported differential traits of OS twins.

## Graphical abstract

1. Sex hormones have prenatal effects on the DNA methylome in OS twins.

2. Prenatal DNA methylomic changes are linked to the nervous system in OS females (OSF), but not OS males (OSM).

3. Prenatal histone modifications differ substantially between OSM and SS males (SSM), but not OSF and SS females (SSF).

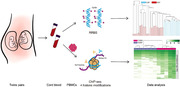

## 1 INTRODUCTION

The incidence of spontaneous dizygotic twinning occurs between 1% and 4%, which might be increased by advanced maternal age and the application of assisted reproductive technologies.^1^ Among the total dizygotic twins, approximately 40% are opposite‐sex (OS).^2^

Androgens, particularly testosterone, serve important functions in early embryonic development.^3^ Testosterone production in fetuses increases from 8 to 24 weeks of gestation and reaches maximal level between 10 and 15 weeks.^4^, ^5^ It overlaps with the period of rapid brain development when microglial cells colonize the cerebrum during 4‐24 gestational weeks.^6^ Twin testosterone transfer hypothesis states that a female having a male co‐twin is exposed to higher levels of prenatal testosterone.^7^ Potential differences in physiological, cognitive, and behavioral traits have been reported between females in OS twins (OSF) and females in SS twins (SSF).^5^ Compared to SSF, OSF may show more masculine traits, such as conservatism,^8^ breaking rules,^9^ poorer performance in high school and college, less likely to marry, reduced fertility, and lower income.^10^ Some studies also suggested no differences between OSF and SSF.^11^, ^12^, ^13^ In contrast to OSF, males in OS twins (OSM) show weak and inconsistent evidence of behavioral and biological changes.^10^, ^14^, ^15^

Animal studies showed that testosterone triggered a DNA demethylation event in mouse embryonic neural stem cells and could affect the global acetylation pattern of histone H3.^16^ Cisternas et al. found that inhibiting DNA methylation in neonatal mice disrupted the testosterone‐dependent masculinization of neurochemical phenotypes.^17^ In addition, human germline cells undergo global DNA demethylation from 7 to 19 weeks, coincident with testosterone production.^18^ Therefore, epigenetic modifications may be a candidate mechanism to explain the more masculine traits in OSF.

To explore the potential influences of prenatal hormone exposure on fetuses, we generated epigenomic data from OS and SS newborn twins. We excluded effects of postnatal environment and socialization so that the epigenetic consequences of prenatal hormones interactions could be investigated exclusively. By comparing DNA methylation and histone modification data, we aim to address how epigenetics may be altered by hormone exposure and may lead to differentiated traits between OSF and SSF, as well as OSM and SSM.

## 2 METHODS AND MATERIALS

### 2.1 Study population and sample collection

A total of 56 families having twins were recruited and then divided into four main categories: OSF, OSM, SSF, and SSM. The twins were born at the Third Hospital of Peking University; an accurate initial medical history confirmed that none suffered birth defects. On the delivery day, ∼1 mL of umbilical cord blood was collected from each twin and processed immediately. Whole‐blood aliquots of 300 μL were used for DNA extraction, and the remaining blood was stored at −80°C for backup. We also collected ∼20 mL blood and extracted cord blood mononuclear cells (CBMCs) as soon as possible.

### 2.2 DNA extraction

The QIAmp DNA Blood Mini Kit (Qiagen, cat.: 51106) was used to extract genomic DNA (gDNA) from the stored whole blood samples.

### 2.3 CBMC extraction and fixation

CBMCs were isolated by Ficoll density‐gradient centrifugation (TBD, cat.: LDS1075).

Freshly‐prepared 1% formaldehyde in 1 × phosphate‐buffered saline (PBS) was used to fix CMBCs for 10 minutes at room temperature. Glycine buffer (125 mmol/L) was added to quench the crosslinking reaction, and the solution was incubated for another 5 minutes at room temperature. The fixed CBMC pellet was cryopreserved at −80°C after washing once with cold 1 × PBS for subsequent chromatin immunoprecipitation sequencing (ChIP‐seq).

### 2.4 Reduced representation bisulfite sequencing

Reduced representation bisulfite sequencing (RRBS) was performed following the published protocol with a few modifications.^19^ In brief, ∼0.3% unmethylated lambda DNA (Thermo Scientific, cat.: SD0021) was spiked into 500 ng of high‐quality gDNA, and the mixture was digested in MspI (Thermo Scientific, cat.: ER0541). The digested DNA was end‐repaired and tailed with deoxyadenosine using Klenow Fragment exo‐ (Thermo Scientific, cat.: EP0422) after purification with Agencourt AMPure XP beads (Agencourt, cat.: A63881). Then NEBNext adapters (NEB, cat.: E7335) were used to ligate overnight with T4 DNA ligase at a high concentration (NEB, cat.: M0202M), and the Uracil‐Specific Excision Reagent enzyme (NEB, cat.: M5505L) was used to digest the products.

Bisulfite conversion was conducted with a MethylCode Bisulfite Conversion Kit (Thermo Scientific, cat.: MECOV‐50). The converted samples were size‐selected by excising gel slices containing 160‐700 bp DNA (2% TAE gel). A gel DNA recovery kit (Vistech, cat.: PC0313) was used to recover and then amplify the converted DNA fragments through PCR with a maximum of 12 cycles with Kapa HiFi U+ Master Mix (Kapa Biosystems, cat.: KK2801). Barcodes were introduced during this process.

The purified PCR‐amplified products were quantified using Qubit dsDNA high‐sensitivity dye in a Qubit 3.0 Fluorometer (Thermo Scientific, cat.: Q33216/Q32854).

A Fragment Analyzer Automated CE System (Analysis Kit: cat.: DNF‐474‐0500) was used to assess the fragment distributions, and a Library Quant Kit for Illumina (NEB, cat.: E7630L) was used to measure molar concentrations. The final qualified libraries were sequenced using the PE150 strategy on an Illumina X Ten sequencer.

### 2.5 ChIP‐seq

ChIP‐seq was performed as previously described.^20^ Briefly, isolated nuclei were sonicated into fragments of 200‐500 bp using an M220 focused‐ultrasonicator (Covaris) with the following parameters: burst, 200; cycle, 20%; and intensity, 8. Sheared chromatin in the supernatant was precleared by co‐incubation with protein A Dynabeads (Invitrogen, cat.: 10002D) at 4°C for 1 hour after centrifugation (13 000 rpm for 10 minutes at 4°C). The supernatant was diluted into 250 μL and transferred to low‐binding Eppendorf tubes. The following antibodies were used for overnight incubation at 4°C with rotation: H3K4me3 (Millipore, cat.: 07‐473), H3K27me3 (Abcam, cat.: ab6002), H3K4me1 (Abcam, cat.: ab8895), and H3K27ac (Abcam, cat.: ab4729). After washing with cold RIPA (10 mM, pH 7.6, HEPES, 1 mM EDTA, 4 mM LiCl, 1% NP‐40, 0.1% N‐lauryl sarcosine) and TEN (1 mM EDTA, 10 mmol/L, pH 8 Tris, 50 mM NaCl), 20 μg of Proteinase K (Qiagen, cat: 19131) were used to digest the immunoprecipitated pellets at 65°C for >6 hours. DNA was purified using a MinElute PCR Purification Kit (Qiagen, cat.: 28006), and libraries were constructed using a NEXTflex ChIP‐Seq Kit (Bioo Scientific, Austin, TX, cat.: 5143‐02). Fragments of 180‐280 bp were size‐selected using Agencourt AMPure XP beads (Beckman, cat.: A63881). DNA fragments were amplified by PCR with 12 cycles after tailing and ligating ChIP‐seq adapters (Bioo Scientific, cat.: 514124). The final library was examined and sequenced as described for RRBS.

### 2.6 RRBS data analysis

Before read mapping, adapters and low‐quality bases in the reads were removed with Trim Galore to optimize paired‐end alignments. The kept reads were aligned to the *Homo sapiens* reference genome (human GRCh38/hg38) using Bismark (version 0.18.1) with the default parameters. The uniquely mapped reads with <2% mismatch were retained for further analyses. Differentially methylated cytosines (DMCs) between any two groups were identified using methylKit with a q‐value threshold of 0.001 and a mean methylation difference of 20%.

In the RRBS samples, the 17.65% (3/17) of SSM and 33.33% (6/18) of SSF are monozygotic twins. In this study, we only focused on the epigenomic characteristics of OS twins (OSF and OSM) by comparing them with SS twins (SSF and SSM). Prenatal hormone exposure of twins is related to the genders of twins, but not zygosity. Thus, from the perspective of hormonal interactions, there are no differences between monozygotic SSF and dizygotic SSF, as well as monozygotic SSM and dizygotic SSM.

### 2.7 ChIP‐seq data analysis

To optimize paired‐end alignment, Trimmomatic was used for trimming before read mapping.^21^ The kept reads were aligned to the *Homo sapiens* reference genome (human GRCh38/hg38) using the Burrows‐Wheeler Aligner with the default parameters in paired‐end mode.^22^ The non‐redundant reads with ≤2% mismatches were retained for further analyses.

The MACS2 peak caller^23^ (version 2.1.0) with the parameter setting “–keep‐dup = 1, –broad” was used to identify peaks of histone modification. The R/Bioconductor package DiffBind was used,^24^ which uses DESeq2 or edgeR to identify differential peak (DPs) with q‐values <.05 and fold changes >2 for H3K4me1, H3K4me3, H3K27ac, and H3K27me3. The shared outputs of the DESeq2 and edgeR methods were taken as the final DPs.

### 2.8 Comparisons in different groups

To study the characteristics of OSF, we compared the Pearson's correlation coefficients of OSF with males (OSM or SSM). Also, we compared OSM with females (OSF or SSF) to clarify the characteristics of OSM. Besides, we also focused on the Pearson's correlation coefficients in the SS twins (females *versus* females: SSF *versus* SSF, OSF *versus* SSF, OSF *versus* OSF; males *versus* males: SSM *versus* SSM, OSM *versus* SSM, OSM *versus* OSM) to further study the characteristics in OSF and OSM. To study the epigenetic effects in OSF, we compared DMCs and histone modifications of OSF *versus* SSF. Meanwhile, we also compared DMCs and histone modifications of OSM *versus* SSM to investigate their potentially epigenetic influences.

### 2.9 Clustering, functional enrichment analysis, and statistics

Hierarchical clustering was used for unsupervised clustering of the epigenomes of OS and SS samples with the R function "*hclust*," in the R base package *stats*. K‐means clustering was used for unsupervised clustering of DMCs using the R function *kcca* with the parameter “family = kccaFamily (which = NULL, dist = distCor)” in the flexclust package. To calculate distance, we used the *dist* function, and for similarity matrices we used the *cor* function of the *stats* package in R with the parameter “method = correlation.” Potential biological functions of genomic regions, such as DMRs and DPs, were determined by GREAT with a p‐value threshold of 10^−5^. For violin box‐plots, the center dot indicated the average; the significance of differences between two groups was determined by the Wilcoxon rank sum test.

## 3 RESULTS

### 3.1 OS twins exhibited distinct DNA methylomic characteristics

**FIGURE 1:**
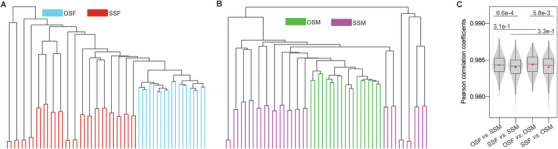
Characterization of genomewide DNA methylation in newborn twins. A and B, Hierarchical clustering for opposite‐sex females (OSF) *versus* same‐sex females (SSF) (A), and opposite‐sex males (OSM) *versus* same‐sex males (SSM) (B) based on DNA methylation level at the top 3% CpG sites with the highest standard deviation. C, Violin‐box plots show the distribution of Pearson's correlation coefficients between genomewide DNA methylations of any two samples from different two groups of twins. The red dots are the arithmetic means. The p‐values between OSF *versus* SSM and SSF *versus* SSM, OSF *versus* OSM and SSF*versus* OSM, OSF*versus* SSM and OSF *versus* OSM, SSF *versus* SSM and SSF *versus* OSM, were shown and determined by Wilcoxon rank‐sum test.

To systematically study the epigenetic differences between OS twins and SS twins, we performed RRBS on cord blood collected from 18 SSF,17 SSM, and 19 OS twins (Tables S1 and S2). We conducted hierarchical clustering on OSF *versus* SSF and OSM *versus* SSM based on the DNA methylation level of top 3% CpG sites with the highest standard deviation. The results showed that samples in OSF *versus* SSF or OSM *versus* SSM were obviously clustered into corresponding subgroups (Figure 1A,B), revealing the different DNA methylation features of OS twins and SS twins with same genders.

**FIGURE 2:**
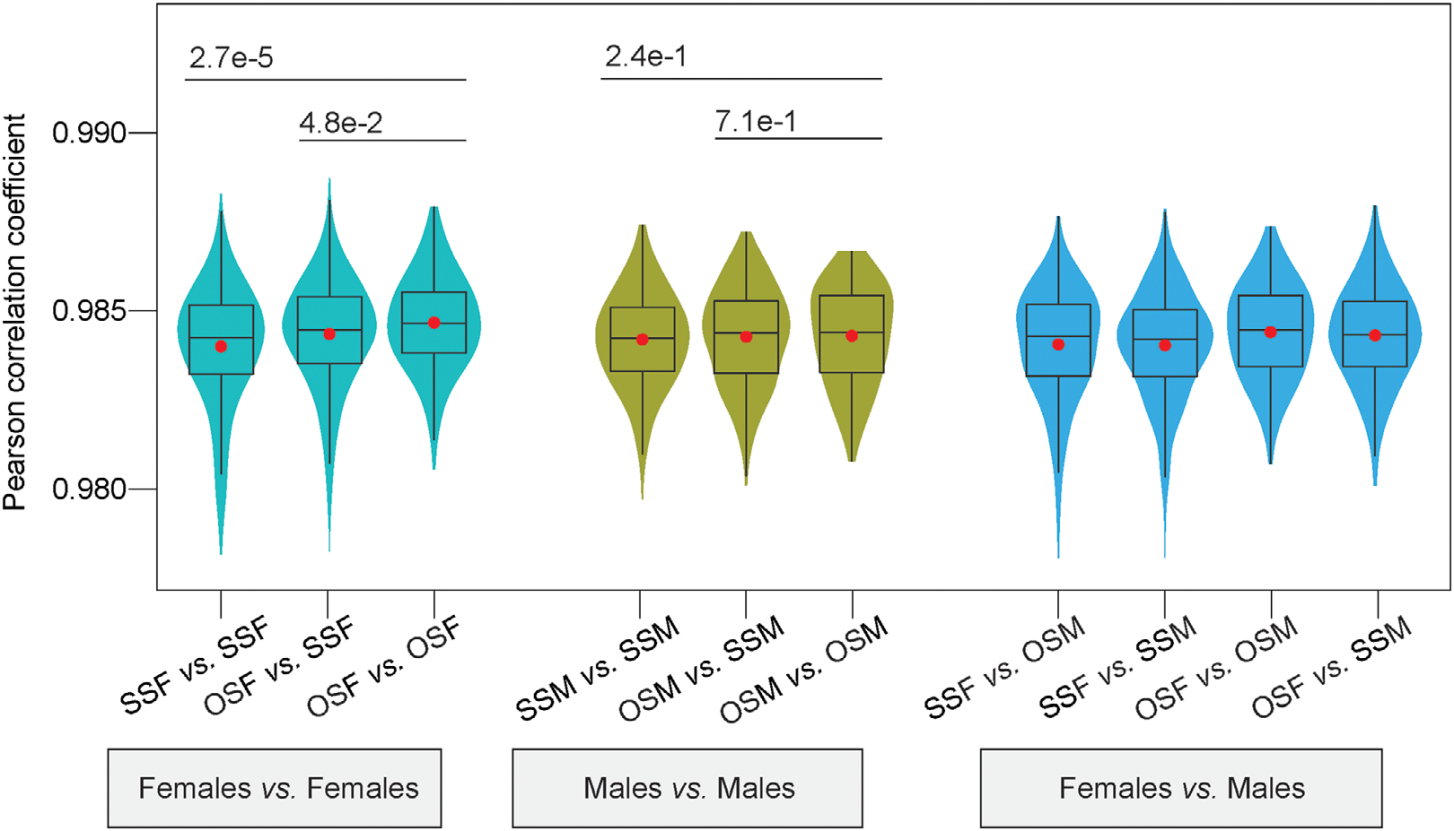
Violin‐box plots show the distribution of Pearson's correlation coefficients between genomewide DNA methylations of any two samples from different two groups of twins. The red dots are the arithmetic means. The p‐values between two groups were shown and determined by Wilcoxon rank‐sum test.

To further identify the distinction between OS twins and SS twins, we performed inter‐group correlation analysis for the four different groups (OSF, OSM, SSF, and SSM), and the comparisons in siblings were excluded. The correlation between OSF and males (OSM or SSM) was significantly higher than the correlation between SSF and males. By contrast, there were no significant differences between the correlation coefficient of "OSM *versus* females (OSF or SSF)" and that of "SSM *versus* females" (Figures 1C and 2). Meanwhile, OSF was more strongly correlated with OSM than with SSM (Figure 1C; Table S3). Furthermore, the intra‐group correlation coefficient of "OSF *versus* OSF" (twin pairs were excluded) was highest among females *versus* females while "OSM *versus* OSM" did not show this characteristic (Figure 2). This indicated that the DNA methylome of OSF became more homogeneous among individuals, potentially shaped by common processes occurring *in utero*.

### 3.2 Different DNA methylation patterns are related to their potential distinct functions in OSF and OSM

**FIGURE 3:**
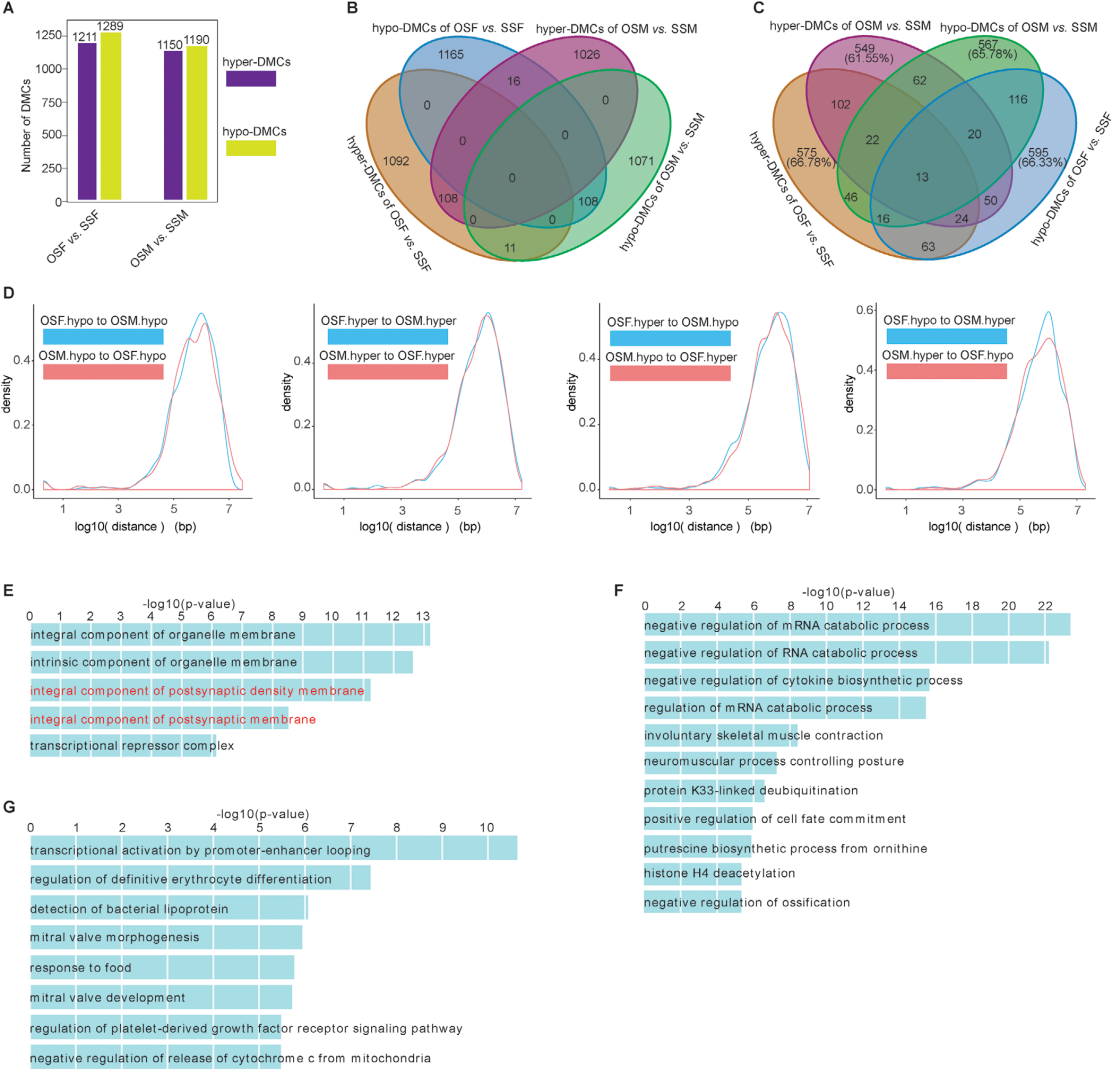
Characterization of DMCs. A, Number of hyper‐ and hypo‐DMCs for OSF*versus* SSF, and OSM *versus* SSM. B, Venn diagrams displayed the overlap among the DMCs. C, Venn diagrams displayed the overlap among the DMCs‐associated genes. D, Distribution of distances between two sets of DMCs. E, Enrichment terms of gene ontology (GO, cellular components) with adjusted p‐value <10^−5^ were shown for the hyper‐DMCs of OSF *versus* SSF. F and G, Enrichment terms of gene ontology (biological process) with adjusted p‐value <10^−5^ were shown for the hyper‐DMCs (F) and hypo‐DMCs (G) of OSM *versus* SSM. The GO terms associated with nervous system were highlighted by red.

**FIGURE 4:**
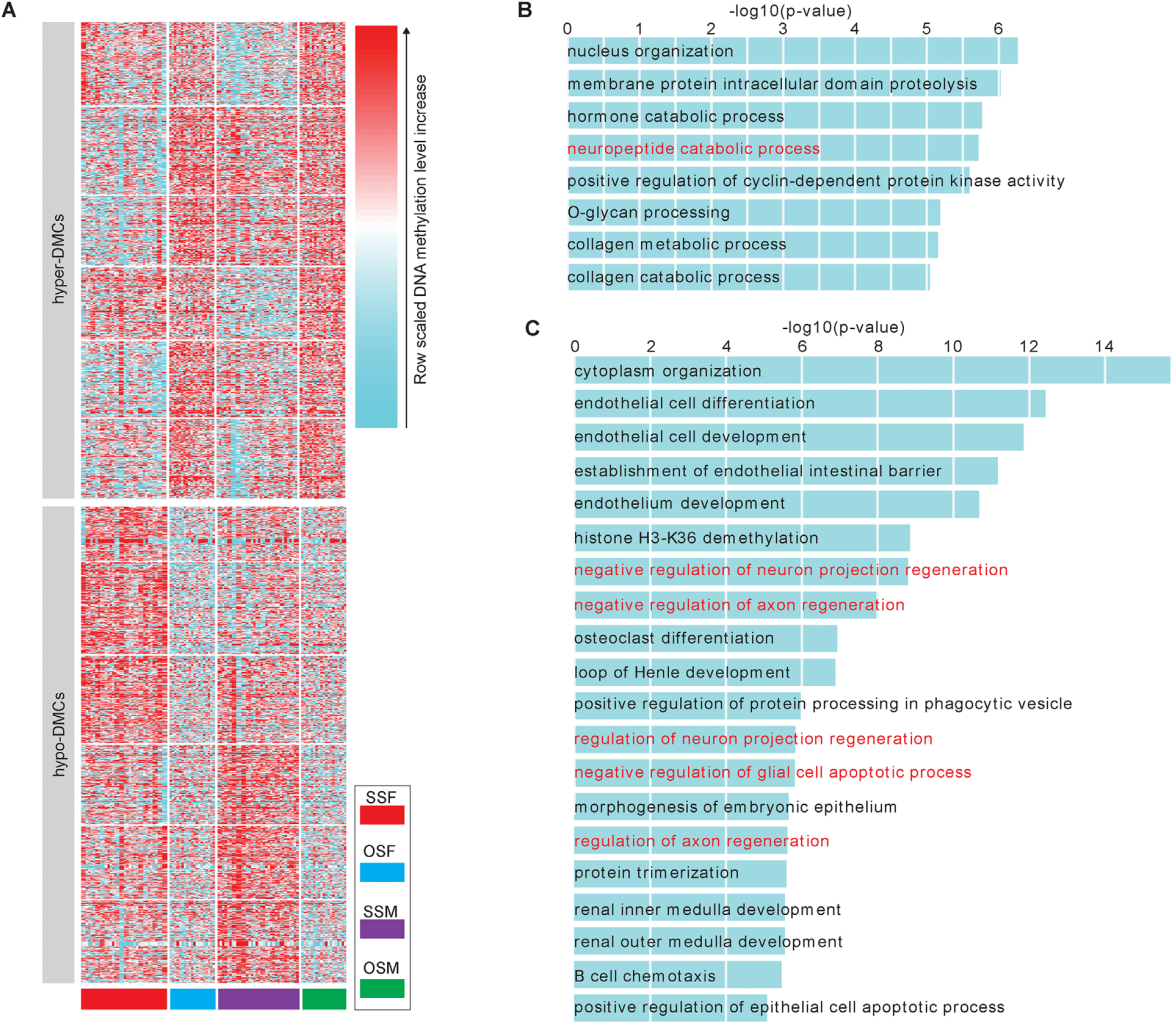
Differentially methylated cytosines (DMCs) for OSF *versus* SSF, and OSM *versus* SSM. A, K‐means clustering for the all merged hyper‐ and hypo‐DMCs. The hyper‐DMCs or hypo‐DMCs for OSF *versus* SSF, and OSM *versus* SSM were merged if they had 1‐bp common region at least, respectively. The hyper‐DMRs (hypo‐DMRs) were classified into six clusters by k‐means clustering on row scaled DNA methylation level. B and C, Enrichment terms of gene ontology (GO, biological process) with adjusted p‐value <10^−5^ were shown for the hyper‐DMCs (B) and hypo‐DMCs (C) of OSF*versus* SSF. The terms associated with nervous system were highlighted by red.

In order to study the potential biological functions of hormone‐induced DNA methylomic changes, we identified DMCs with q‐values <0.001 and methylation differences >20% in "OSF *versus* SSF," and "OSM *versus* SSM." The results showed that there were ∼1000 DMCs in these two comparisons. Between the two comparisons, only 10% (108) of hypo methylated cytosines (hypo‐DMCs) and 10% (108) of hyper methylated cytosines (hyper‐DMCs) were overlapped (Figure 3A and B; Table S4). To further determine whether OSF and OSM induced same methylation changes, we performed cluster analysis based on DNA methylation levels of merged hyper‐DMCs and hypo‐DMCs in all samples. The data showed that for both hyper‐DMCs and hypo‐DMCs, no consistent patterns of DNA methylation levels were present in any cluster of OSF and OSM (Figure 4A). In addition, the overlapping proportion of DMCs associated genes was less than 20% (Figure 3C); the genomic distances between DMCs in OSF and OSM were mostly more than 100 kb (Figure 3D). These results suggested that the induced changes in DNA methylation and their functions might be very different in OSF and OSM.

To identify the potential impacts of those specific alterations in OSF and OSM, we performed gene ontology enrichment analysis and found that hyper‐DMCs of "OSF *versus* SSF" were significantly enriched in nervous system and catabolic process, such as neuropeptide catabolic process, hormone catabolic process, and collagen catabolic process. Comparatively, hypo‐DMCs of "OSF *versus* SSF" were also mainly involved in nervous system regulation, such as negative regulation of neuron projection regeneration, negative regulation of axon regeneration, and regulation of integral component of postsynaptic membrane (Figures 3E and 4B,C; Tables S5 and S6). Therefore, the differential DNA methylation in OSF might mainly affect regulation and catabolic processes of the nervous system. Comparatively, DNA methylation changes in OSM affected RNA catabolic process and neuromuscular process controlling posture (Figure 3F,G; Tables S7 and S8).

### 3.3 Differential histone modifications are more observed in "OSM *versus* SSM" group than "OSF *versus* SSF" group

**FIGURE 5:**
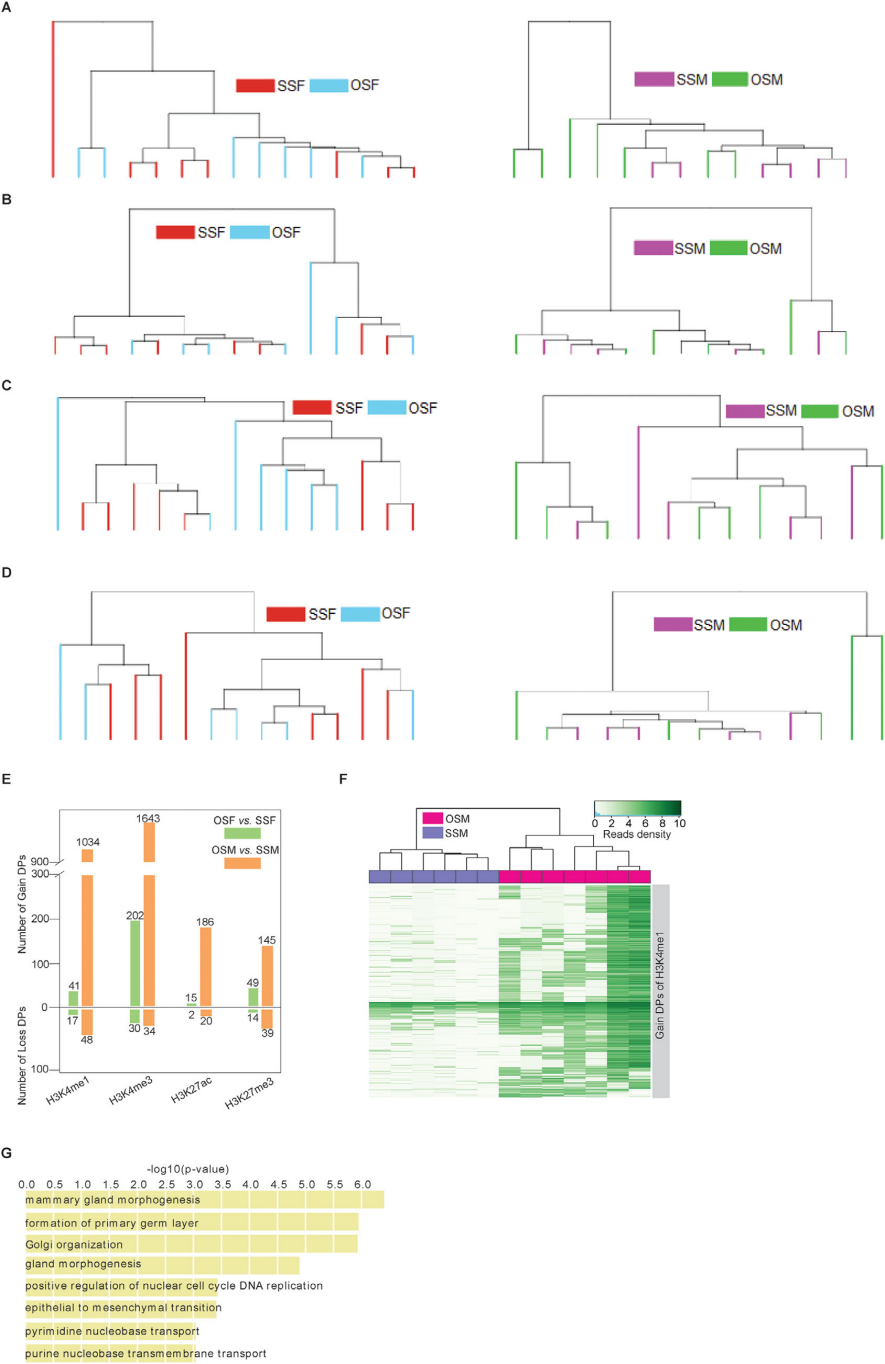
Histone modifications and their differences in twins. A‐D, Hierarchical clustering for females (left) and males (right) based on their H3K4me1 (A), H3K4me3 (B), H3K27ac (C), and H3K27me3 (D) level. E, Number of differential peaks of histone modifications. F, Hierarchical clustering for OSM and SSM twins based on H3K4me1 level at the H3K4me1 DPs of OSM *versus* SSM. G, Enrichment terms of gene ontology (biological process) with adjusted p‐value <10^−5^ were shown for the 1034 GAIN H3K4me1 DPs of OSM *versus* SSM.

**FIGURE 6:**
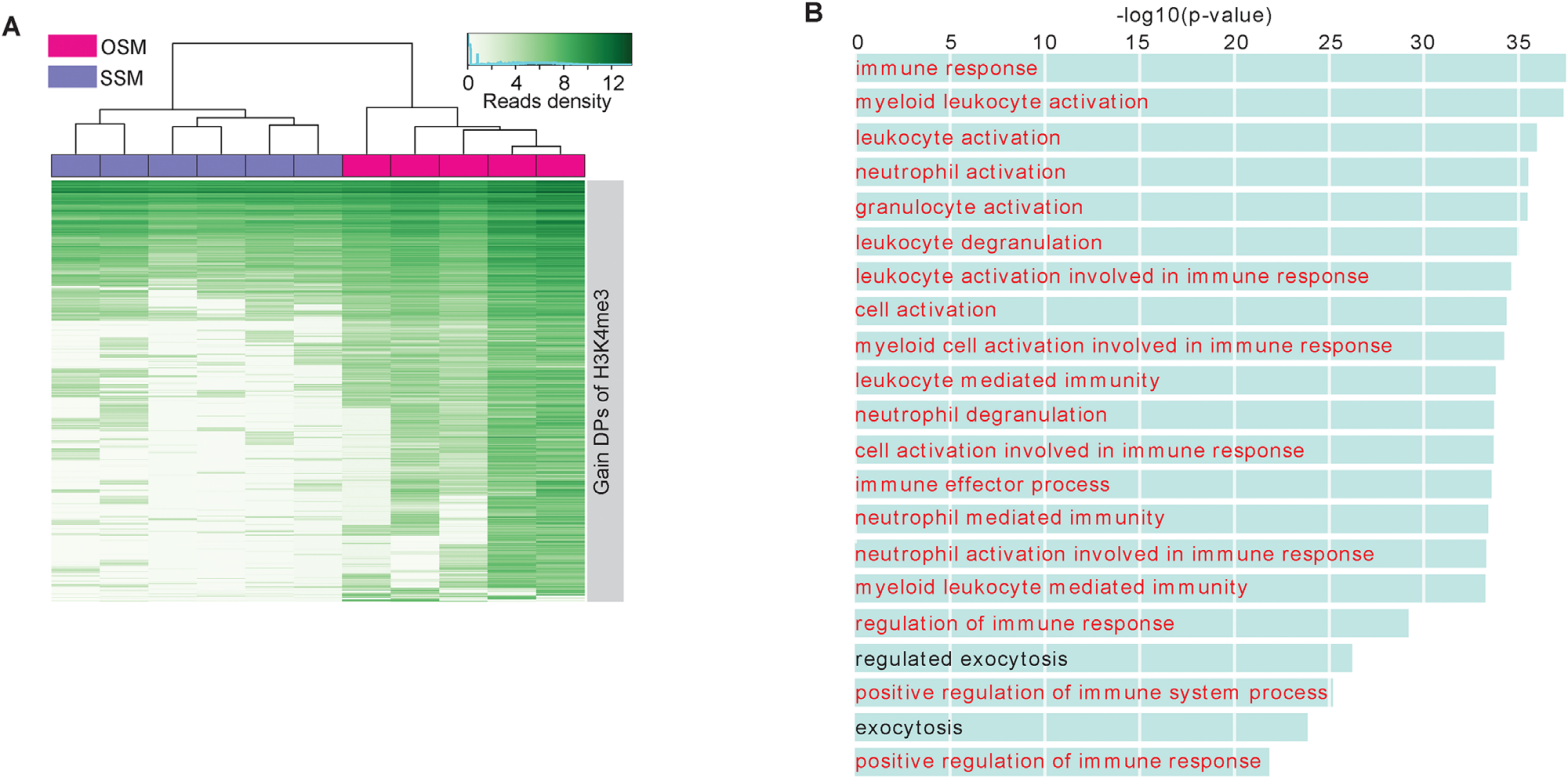
H3K4me1 and H3K4me3 differential peaks (DPs) for OSM *versus* SSM. A, Hierarchical clustering for OSM and SSM twins based on H3K4me3 level at the H3K4me3 DPs of OSM *versus* SSM. B, Enrichment terms of gene ontology (biological process) with adjusted p‐value <10‐5 were shown for the 1643 GAIN H3K4me3 DPs of OSM versus SSM. The GO terms associated with immune system were highlighted by red.

**FIGURE 7:**
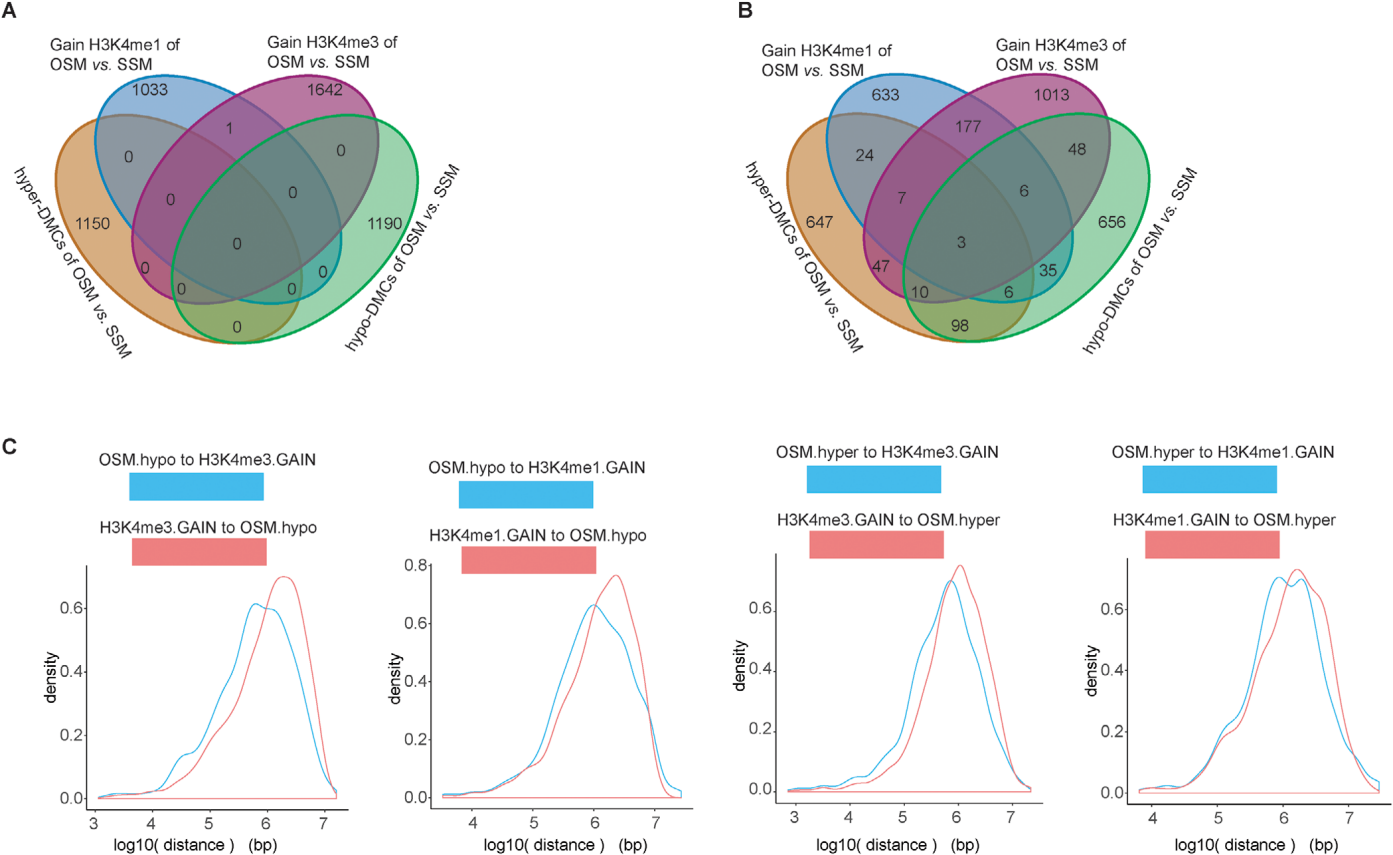
Correlation between DPs and DMCs. A, Venn diagrams displayed the overlap among the DPs and DMCs. B, Venn diagrams displayed the overlap among the DPs and DMCs‐associated genes. C, Distribution of distances between the two sets, DMCs and DPs.

In addition to DNA methylome, histone modifications may also be altered by the environment *in utero*.^25^, ^26^ Therefore, we used ChIP‐seq in core blood mononuclear cells collected from seven OS twins, four SSF twins, and three SSM twins to examine changes in histone modifications, including H3K4me1, H3K4me3, H3K27ac, and H3K27me3. Based on genome‐wide histone modifications, neither the "OSF *versus* SSF" group nor the "OSM *versus* SSM" group could be clearly identified and separated in hierarchical clustering analysis (Figure 5A‐D; Tables S9 and S10). Differential peak (DP) analysis revealed that more than 1000 GAIN H3K4me1 DPs and GAIN H3K4me3 DPs were identified in "OSM *versus* SSM," while only a few GAIN/LOSS DPs could be identified in "OSF *versus* SSF" (Figure 5E). Through GAIN DPs of H3K4me1, OSM, and SSM could be clearly distinguished (Figure 5F). Furthermore, functional enrichment analysis showed that GAIN DPs of H3K4me1 between "OSM versus SSM" were enriched in the processes of gland morphogenesis, DNA replication, and transport (Figure 5G; Table S11). In addition to H3K4me1, OSM and SSM could also be separated by GAIN DPs of H3K4me3 (Figure 6A), which were highly enriched in immune response (Figure 6B; Table S11). We also focused on further analyzing the relationship between DMCs and DPs. Almost no overlaps in genomic regions between DMCs and DPs were observed, along with only a few DMC‐ and DP‐associated genes (including GAIN H3K4me1 and GAIN H3K4me3) overlapped among all groups (Figure 7A,B). The genomic distances between DMCs and DPs (including GAIN H3K4me1 and GAIN H3K4me3) were mostly more than than 100 kb (Figure 7C).

## 4 DISCUSSION

Prenatal hormone interactions in OS twins may explain their different phenotypic traits in some clinical cohorts, while the underlying mechanism is still unclear. In this study, we recruited 56 pairs of twins (OS and SS twins) to exclude potential confounders such as postnatal environmental and social factors. For the first time, the epigenetic consequences of prenatal hormone interactions were investigated. We systematically explored the differences in genome‐wide DNA methylation changes and histone modifications between OS and SS twins.

From DNA methylome data, OSF presented different characteristics compared to SSF. "OSF *versus* males (OSM or SSM)" showed stronger correlation than "SSF *versus* males," suggesting that OSF tended to be more masculine and might become closer to their OSM brothers in DNA methylome. In contrast, OSM and SSM did not have significant differences when comparing their correlations with OSF and SSF. Therefore, OSM may be less influenced by their female co‐twins according to the DNA methylation data.

We further investigated the potential functions of DNA methylome changes in OSF. Analysis of DMCs showed methylation changes were associated with nervous system development and regulation, which might explain the reported traits about differential perceptual and cognitive in OSF. In two cohort studies, OSF between 20 and 38 months tended to be somewhat weak in expressive vocabulary and scored lower on the MacArthur Communicative Developmental Inventory than SSF.^27^, ^28^ OSF also performed better than SSF twins in visuospatial cognition assessed by the Mental Rotation Test.^29^, ^30^

Although DMC‐related genes were only correlated with nervous system functions in OSF, both OSF and OSM presented a pronounced enrichment of DMC‐related genes involved in catabolic processes. A Swedish cohort study of twins (>60 years old) reported that OSF had a moderately higher body mass index, body weight, and rate of dyslipidemia than SSF.^31^ The phenotypes and associated potential risks need further attention.

In addition to DNA methylation, epigenetic histone modification may regulate chromatin status and gene expression.^32^ Previous studies were mainly focused on the epigenetics of monozygotic twins to study the cause for some types of phenotypic discordance between monozygotic twin pairs.^33^ Our study offers some insights on histone modification differences between OSM and SSM. We found that DPs of H3K4me1 and H3K4me3 could be used to distinguish OSM from SSM; yet none of the four histone modifications could distinguish OSF from SSF. Interestingly, we found that differential H3K4me3 modifications in "OSM *versus* SSM" might be related to immune responses. A Danish cohort study showed lower early‐life mortality risks in OSM than SSM.^34^

When evaluating the effects of epigenetic modifications in OS twins, age may be a critical factor.^35^, ^36^, ^37^, ^38^ An Australian study using two cohorts (younger cohort and older cohort) reported that masculine characteristics (OSF scoring higher on rule‐breaking than SSF) only occurred in the younger cohort (mean = 23.2), while the older cohort (mean = 41.2) showed no significant differences.^9^ Another study from Denmark and Sweden showed no significant differences in the incidence rate ratios of cancers between OS and SS twins.^39^ Previous studies were mainly focused on adult twins and may therefore obtain inconsistent results. Our study used newborns, which excluded postnatal environmental and age as potential confounders, so that the hormone effects of OS twins *in utero* could be investigated exclusively. However, our study also has some limitations. The sample size was not very large compared to clinical cohort studies. We would need more clinical samples to verify our results. In the same‐sex twins, we didn't differentiate their zygosity (including monozygotic twins and dizygotic twins). It may be an influence factor when we performed inter‐group comparing analysis between SS twins and OS twins. The specific biases and differences introduced by monozygotic or dizygotic twins need further investigation in a larger cohort considering the small portion of monozygotic twins in our study. The postnatal follow‐up data should also be analyzed in the future to further confirm the epigenetic changes and to investigate the effects of age. However, for those traits that may only appear long after, the phenotypes could also be strongly impacted by environmental factors which may also be a confounding factor.

In conclusion, we provide an epigenetic basis to the understanding of hormonal interactions in OS twins. Our results highlighted that DNA methylation of OSF was significantly influenced by their male co‐twins in newborns with potential impacts on nervous system development and regulation. DMCs involved in catabolic processes are prone to be affected in both OSF and OSM, and immune responses in OSM might be affected through histone modifications. Long‐term follow‐up is necessary to provide more solid and insightful evidence.

## CONFLICT OF INTEREST

The authors declare that there is no conflict of interest that could be perceived as prejudicing the impartiality of the research reported.

## ETHICAL APPROVAL

All blood samples were obtained after participants have given written informed consent and were fully anonymized (each individual was given a code). The Reproductive Study Ethics Committee of Peking University Third Hospital approved the study (approved protocol no. 201752‐044 in 2017/06/20). All relevant ethical regulations were followed.

## AUTHOR CONTRIBUTIONS

Siming Kong, Yong Peng, Wei Chen, and Liying Yan wrote the manuscript. Wei Chen and Xinyi Ma collected the study materials, samples, and patient data, and performed the experiments. Yong Peng developed the analysis methods and performed bioinformatics analysis. Jie Qiao, Liying Yan, and Wei Chen developed the experimental concept and design. All the authors read and approved the final manuscript.

## CODE AVAILABILITY

The bioinformatics pipelines and scripts used for our analysis are available at https://github.com/CTLife/Opposite-Sex-Twins_Epigenome.

## Supporting information




**Supplementary_Table_1**:The samples, number of reads, and mapping ratio for RRBS and ChIP‐seq data were summarized

**Supplementary_Table_2**:Clinical data for RRBS samples

**Supplementary_Table_3**:The list of Pearson correlation coefficients of DNA methylation level between any two RRBS samples

**Supplementary_Table_4**:The list of the hyper‐ and hypo‐DMCs for OSF versus SSF, and OSM versus SSM

**Supplementary_Table_5**:For Figure 2b, the enrichment gene ontology (biological process) terms for the hyper‐DMCs of OSF versus SSF

**Supplementary_Table_6**:For Figures S2C and E, the enrichment gene ontology (biological process or cellular component) terms for the hypo‐DMCs of OSF versus SSF

**Supplementary_Table_7**:For Figure S2F, the enrichment gene ontology (biological process) terms for the hyper‐DMCs of OSM versus SSM

**Supplementary_Table_8**:For Figure S2G, the enrichment gene ontology (biological process) terms for the hypo‐DMCs of OSM versus SSM

**Supplementary_Table_9**:The list of the gain and loss differential peaks (DPs) of H3K4me1, H3K4me3, H3K27ac, and H3K27me3 for OSF versus SSF

**Supplementary_Table_10**:The list of the gain and loss DPs of H3K4me1, H3K4me3, H3K27ac, and H3K27me3 for OSM versus SSM

**Supplementary_Table_11**:For Figures 3B and 3D, the enrichment gene ontology (biological process) terms for the H3K4me1 and H3K4me3 Gain DPs of OSM versus SSM

Supporting Information

## Data Availability

All the raw Data included in this study have been uploaded to the Sequence Read Archive database (https://trace.ncbi.nlm.nih.gov/Traces/home/) and are available for download via accession number GSE136849.

## References

[1] Mbarek H , Steinberg S , Nyholt DR , et al. Identification of common genetic variants influencing spontaneous dizygotic twinning and female fertility. Am J Hum Genet. 2016;98(5):898‐908. 27132594 10.1016/j.ajhg.2016.03.008 PMC4863559

[2] Kanazawa S , Segal NL , de Meza D . Why are there more same‐sex than opposite‐sex dizygotic twins?. Hum Reprod. 2018;33(5):930‐934. 29534175 10.1093/humrep/dey046

[3] Hines M . Gender development and the human brain. Annu Rev Neurosci. 2011;34:69‐88. 21438685 10.1146/annurev-neuro-061010-113654

[4] Word RA , George FW , Wilson JD , Carr BR . Testosterone synthesis and adenylate cyclase activity in the early human fetal testis appear to be independent of human chorionic gonadotropin control. J Clin Endocrinolo Metab. 1989;69(1):204‐208. 10.1210/jcem-69-1-204 2732296

[5] Ahrenfeldt LJ , Christensen K , Segal NL , Hur YM . Opposite‐sex and same‐sex twin studies of physiological, cognitive and behavioral traits. Neurosci Biobehav Rev. 2020;108:322‐340. 31711815 10.1016/j.neubiorev.2019.11.004 PMC6949417

[6] Menassa DA . Gomez‐Nicola D . Microglial dynamics during human brain development. Front Immunol. 2018;9:1014. 29881376 10.3389/fimmu.2018.01014 PMC5976733

[7] Miller EM . Prenatal sex hormone transfer: a reason to study opposite‐sex twins. Pers Individ Dif. 1994;17(4):511‐529.

[8] Miller EM , Martin N . Analysis of the effect of hormones on opposite‐sex twin attitudes: twin research. Acta Genet Med Gemellol. 1995;44(1):41‐52. 7653203 10.1017/s0001566000001884

[9] Loehlin JC , Martin NG . Dimensions of psychological masculinity‐femininity in adult twins from opposite‐sex and same‐sex pairs. Behav Genet. 2000;30(1):19‐28. 10934796 10.1023/a:1002082325784

[10] Butikofer A , Figlio DN , Karbownik K , Kuzawa CW , Salvanes KG . Evidence that prenatal testosterone transfer from male twins reduces the fertility and socioeconomic success of their female co‐twins. Proc Natl Acad Sci U S A. 2019;116(14):6749‐6753. 30886089 10.1073/pnas.1812786116 PMC6452670

[11] Ho A , Todd RD , Constantino JN . Brief report: autistic traits in twins vs. non‐twins—a preliminary study. J Autism Dev Disord. 2005;35(1):129‐133. 15796128 10.1007/s10803-004-1040-8

[12] Lenz B , Muller CP , Kornhuber J . Alcohol dependence in same‐sex and opposite‐sex twins. J Neural Transm (Vienna). 2012;119(12):1561‐1564. 23104613 10.1007/s00702-012-0907-7

[13] Korsoff P , Bogl LH , Korhonen P , et al. A comparison of anthropometric, metabolic, and reproductive characteristics of young adult women from opposite‐sex and same‐sex twin pairs. Front Endocrinol (Lausanne). 2014;5:28. 24639667 10.3389/fendo.2014.00028 PMC3945783

[14] Peper JS , Brouwer RM , van Baal GCM , et al. Does having a twin brother make for a bigger brain?. Eur J Endocrinol. 2009;160(5):739‐746. 19218283 10.1530/EJE-08-0915

[15] Marečková K , Chakravarty MM , Lawrence C , et al. Identifying craniofacial features associated with prenatal exposure to androgens and testing their relationship with brain development. Brain Struct Funct. 2015;220(6):3233‐3244. 25074752 10.1007/s00429-014-0852-3

[16] Bramble MS , Roach L , Lipson A , et al. Sex‐specific effects of testosterone on the sexually dimorphic transcriptome and epigenome of embryonic neural stem/progenitor cells. Sci Rep. 2016;6(1):1‐13. 27845378 10.1038/srep36916 PMC5109279

[17] Cisternas CD , Cortes LR , Golynker I , Castillo‐Ruiz A , Forger NG . Neonatal inhibition of DNA methylation disrupts testosterone‐dependent masculinization of neurochemical phenotype. Endocrinology. 2020;161(1):bqz022. 31742329 10.1210/endocr/bqz022

[18] Wen L , Tang F . Human germline cell development: from the perspective of single‐cell sequencing. Mol Cell. 2019;76(2):320‐328. 31563431 10.1016/j.molcel.2019.08.025

[19] Gu H , Smith ZD , Bock C , Boyle P , Gnirke A , Meissner A . Preparation of reduced representation bisulfite sequencing libraries for genome‐scale DNA methylation profiling. Nat Protoc. 2011;6(4):468‐481. 21412275 10.1038/nprot.2010.190

[20] Wang Y , Li Y , Guo C , et al. ISL1 and JMJD3 synergistically control cardiac differentiation of embryonic stem cells. Nucleic Acids Res. 2016;44(14):6741‐6755. 27105846 10.1093/nar/gkw301 PMC5001586

[21] Bolger AM , Lohse M , Usadel B . Trimmomatic: a flexible trimmer for Illumina sequence data. Bioinformatics. 2014;30(15):2114‐2120. 24695404 10.1093/bioinformatics/btu170 PMC4103590

[22] Li H , Durbin R . Fast and accurate long‐read alignment with Burrows‐Wheeler transform. Bioinformatics. 2010;26(5):589‐595. 20080505 10.1093/bioinformatics/btp698 PMC2828108

[23] Zhang Y , Liu T , Meyer CA , et al. Model‐based analysis of ChIP‐SEquation (MACS). Genome Biol. 2008;9(9):R137. 18798982 10.1186/gb-2008-9-9-r137 PMC2592715

[24] Ross‐Innes CS , Stark R , Teschendorff AE , et al. Differential oestrogen receptor binding is associated with clinical outcome in breast cancer. Nature. 2012;481(7381):389‐393. 22217937 10.1038/nature10730 PMC3272464

[25] Tang B , Jia H , Kast RJ , Thomas EA . Epigenetic changes at gene promoters in response to immune activation in utero. Brain Behav Immun. 2013;30:168‐175. 23402795 10.1016/j.bbi.2013.01.086

[26] Li Y , Saldanha SN , Tollefsbol TO . Impact of epigenetic dietary compounds on transgenerational prevention of human diseases. AAPS J. 2014;16(1):27‐36. 24114450 10.1208/s12248-013-9538-7 PMC3877417

[27] Galsworthy MJ , Dionne G , Dale PS , Plomin R . Sex differences in early verbal and non‐verbal cognitive development. Dev Sci. 2000;3(2):206‐215.

[28] Hulle CAV , Goldsmith HH , Lemery KS . Genetic, environmental, and gender effects on individual differences in toddler expressive language. J Speech Lang Hear Res. 2004;47(4):904‐913. 15324294 10.1044/1092-4388(2004/067)

[29] Vuoksimaa E , Kaprio J , Kremen WS , et al. Having a male co‐twin masculinizes mental rotation performance in females. Psychol Sci. 2010;21(8):1069‐1071. 20581340 10.1177/0956797610376075 PMC4438761

[30] Heil M , Kavšek M , Rolke B , Beste C , Jansen P . Mental rotation in female fraternal twins: evidence for intra‐uterine hormone transfer?. Biol Psychol. 2011;86(1):90‐93. 21094200 10.1016/j.biopsycho.2010.11.002

[31] Alexanderson C , Henningsson S , Lichtenstein P , Holmäng A , Eriksson E . Influence of having a male twin on body mass index and risk for dyslipidemia in middle‐aged and old women. Int J Obes. 2011;35(12):1466‐1469. 10.1038/ijo.2011.18 21386807

[32] Esteller M . Epigenetics in cancer. N Engl J Med. 2008;358(11):1148‐1159. 18337604 10.1056/NEJMra072067

[33] Fraga MF , Ballesta E , Paz MF , et al. Epigenetic differences arise during the lifetime of monozygotic twins. Proc Natl Acad Sci. 2005;102(30):10604‐10609. 16009939 10.1073/pnas.0500398102 PMC1174919

[34] Ahrenfeldt LJ , Larsen LA , Lindahl‐Jacobsen R , et al. Early‐life mortality risks in opposite‐sex and same‐sex twins: a Danish cohort study of the twin testosterone transfer hypothesis. Ann Epidemiol. 2017;27(2):115‐120.e2. 28024904 10.1016/j.annepidem.2016.11.011 PMC5439218

[35] Rakyan VK , Down TA , Maslau S , et al. Human aging‐associated DNA hypermethylation occurs preferentially at bivalent chromatin domains. Genome Res. 2010;20(4):434‐439. 20219945 10.1101/gr.103101.109 PMC2847746

[36] Florath I , Butterbach K , Muller H , Bewerunge‐Hudler M , Brenner H . Cross‐sectional and longitudinal changes in DNA methylation with age: an epigenome‐wide analysis revealing over 60 novel age‐associated CpG sites. Hum Mol Genet. 2014;23(5):1186‐1201. 24163245 10.1093/hmg/ddt531 PMC3919014

[37] McClay JL , Aberg KA , Clark SL , et al. A methylome‐wide study of aging using massively parallel sequencing of the methyl‐CpG‐enriched genomic fraction from blood in over 700 subjects. Hum Mol Genet. 2014;23(5):1175‐1185. 24135035 10.1093/hmg/ddt511 PMC3919012

[38] Steegenga WT , Boekschoten MV , Lute C , et al. Genome‐wide age‐related changes in DNA methylation and gene expression in human PBMCs. Age (Dordr). 2014;36(3):9648. 24789080 10.1007/s11357-014-9648-x PMC4082572

[39] Ahrenfeldt LJ , Skytthe A , Moller S , et al. Risk of sex‐specific cancers in opposite‐sex and same‐sex twins in Denmark and Sweden. Cancer Epidemiol Biomarkers Prev. 2015;24(10):1622‐1628. 26282631 10.1158/1055-9965.EPI-15-0317 PMC4782008

