# Supplementary material for: Epigenetic consequences of hormonal interactions between opposite‐sex twin fetuses
Source: Clin Transl Med. 2020 Dec 4;10(8):e234. doi: 10.1002/ctm2.234 (PMC7717068; doi:10.1002/ctm2.234)
Supplement: Supplementary file 12 — Supporting Information [file CTM2-10-e234-s012.docx]

**Supplementary_Table_1：**The samples, number of reads and mapping ratio for RRBS and ChIP-seq data were summarized.

**Supplementary_Table_2：**Clinical data for RRBS samples.

**Supplementary_Table_3：**The list of Pearson correlation coefficients of DNA methylation level between any two RRBS samples.

**Supplementary_Table_4：**The list of the hyper- and hypo-DMCs for OSF vs SSF, and OSM vs SSM.

**Supplementary_Table_5：**For fig. 2b, the enrichment gene ontology (biological process) terms for the hyper-DMCs of OSF vs SSF.

**Supplementary_Table_6：**For fig. 2c and S2e, the enrichment gene ontology (biological process or cellular component) terms for the hypo-DMCs of OSF vs SSF.

**Supplementary_Table_7：**For fig. S2f, the enrichment gene ontology (biological process) terms for the hyper-DMCs of OSM vs SSM.

**Supplementary_Table_8：**For fig. S2g, the enrichment gene ontology (biological process) terms for the hypo-DMCs of OSM vs SSM.

**Supplementary_Table_9：**The list of the gain and loss differential peaks (DPs) of H3K4me1, H3K4me3, H3K27ac and H3K27me3 for OSF vs SSF.

**Supplementary_Table_10：**The list of the gain and loss DPs of H3K4me1, H3K4me3, H3K27ac and H3K27me3 for OSM vs SSM.

**Supplementary_Table_11：**For fig. 3b and 3d, the enrichment gene ontology (biological process) terms for the H3K4me1 and H3K4me3 Gain DPs of OSM vs SSM.
